# Association analysis of *SYT11*, *FGF20*, *GCH1* rare variants in Parkinson's disease

**DOI:** 10.1111/cns.13745

**Published:** 2021-10-21

**Authors:** Jia‐li Pu, Zhi‐Hao Lin, Ran Zheng, Yi‐Qun Yan, Nai‐jia Xue, Xin‐zhen Yin, Bao‐Rong Zhang

**Affiliations:** ^1^ Department of Neurology Second Affiliated Hospital College of Medicine Zhejiang University Hangzhou China

## CONFLICT OF INTEREST

The authors declare no conflicts of interest.

Dear editors,

Mounting evidence has demonstrated that the genetic factors play an important role in PD (Parkinson's disease). Previous studies have identified several genetic variants that are associated with Parkinson's disease in eastern China.^1^, ^2^ Recently, Uladzislau Rudakou and colleagues identified a rare 3′ UTR variant (rs945006601), a rare 5′ UTR variant (rs1034608171), the rare nonsynonymous variants that drive the *SYT11*, *FGF20*, *GCH1* associated with PD in three cohorts, respectively.^3^ In addition, they found that common variants in *MAPT*, *TMEM175*, *BST1*, *SNCA*, and *GPNMB* are linked with PD. Since race is an important factor for the genetic association test, we investigated these rare variants in *SYT11*, *FGF20*, and *GCH1* genes in our eastern Chinese Han population.

In our study, 516 patients with sporadic PD (mean age: 61.35 ± 10.56; male/female: 289/227) and 498 ethnicity‐, age‐, and sex‐matched healthy controls (mean age: 60.14 ± 11.44; male/female: 287/211) were included by two movement disorder specialists according to Movement Disorder Society clinical diagnostic criteria in the second affiliated hospital of Zhejiang University of Medicine (Hangzhou, China).^4^ All participants have provided written informed consent. The ethics of this study was approved by the ethics committee of the second affiliated hospital of Zhejiang University of Medicine.

All blood samples were collected, and then genomic DNA was extracted from peripheral blood leucocytes using QIAamp DNA Blood Mini Kit (Qiagen) according to the manufacturer's protocol. We sequenced these rare variants using the MassArray and KASP assays, and the details of the primers were provided in Tables S1 and S2. The results were analyzed using a MassARRAY Analyzer (Agena Bioscience) and an ABI 7900 Analyzer (Applied Biosystems), respectively. We used Pearson's chi‐square test to compare allelic frequencies of these 10 rare variants between PD and healthy controls. All statistical analyses were conducted using SPSS20.0 (IBM). We predicted the pathogenicity of the variant (position: 55312562; c.C552T, p.R186C) by CADD, Polyphen2, SIFT, MutationTaster in VarCards website (http://varcards.biols.ac.cn/). The threshold values were set for the harmfulness of the variants: CADD>15; Polyphen2 > 0.86; SIFT<0.05.

**FIGURE 1:**
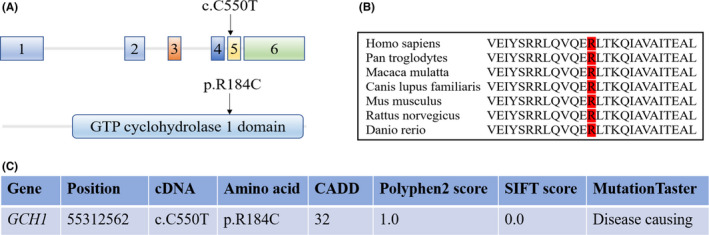
Schematic of human GCH1 and pathogenetic analysis of c.C552T, p.R186C variant. (A) The c.C552T, p.R186C variant locates in *GCH1* gene and GCH1 protein. (B) The position and the surrounding sequence of GCH1 p.R186C are highly conserved across different species. (C) Pathogenicity prediction of c.C552T, p.R186C variant in silico. Threshold values for deleteriousness: CADD greater than 15; Polyphen2 greater than 0.86; SIFT less than 0.05 [Colour figure can be viewed at wileyonlinelibrary.com]

In our cohort, we did not find any significantly different rare variants between PD patients and healthy controls reported by Uladzislau Rudakou. One nonsynonymous variant (position: 55312562; c.C552T, p.R186C) in the *GCH1* gene was found in eight PD patients and four healthy controls (Table 1). The p.R186C variant is located in the GTP cyclohydrolase 1 domain and is highly conserved across different species (Figure 1), suggesting that the p.R186C variant might change the function of GCH1 and contribute to the pathogenesis of PD. Moreover, all pathogenicity predictions in silico (CADD, Polyphen2, SIFT, MutationTaster) indicated that the p.R186C variant was harmful to PD (Figure 1). However, there is no statistically significant difference between PD patients and healthy controls in our cohort.

**TABLE 1:** Variants of *SYT11*, *FGF20*, and *GCH1* genes identified in our cohort

Gene	Position	dbSNP ID	Ref/Alt	PD	Control	*p*	OR (95% CI)
*SYT11*	155852207	rs945006601	G/C	1030/0	998/0		
*FGF20*	16859615	rs1034608171	G/C	1032/0	998/0		
*GCH1*	55312498	NA	A/T	1032/0	1000/0		
	55312559	NA	G/C	1032/0	1000/0		
	55312562	NA	G/A	1012/8	986/4	0.269	1.949 (0.585–6.492)
	55332087	NA	C/T	1032/0	1000/0		
	55310826	rs104894434	A/G	1032/0	998/0		
	55312502	rs200891969	C/T	1012/0	986/0		
	55310817	rs41298442	T/C	1020/0	992/0		
	55326454	rs756256944	C/T	1032/0	996/0		

Abbreviations: Alt, alternate allele; OR, odds ratio; PD, Parkinson's disease; Ref, reference allele.

The SYT11 protein, synaptotagmin‐11, is a synaptotagmin isoform, which regulates membrane trafficking in synaptic transmission.^5^ Genetic studies have reported that the mutations in the SYT11 gene were linked with PD and schizophrenia.^6^, ^7^ Previous studies have suggested that the variants in the nearby GBA gene drive the SYT11 GWAS association with PD.^8^ However, after exclusion of all GBA variant carriers, Uladzislau Rudakou and colleagues found that the rs945006601 in the *SYT11* gene remained statistically significant between PD patients and healthy controls. Therefore, the variant rs945006601 in the *SYT11* gene linked with PD was independent of *GBA* variants. Conversely, in our eastern Chinese Han population, we did not find rs945006601 which likely indicated the important effect of ethnic context in *SYT11* variants.

The protein encoded by *FGF20*, fibroblast growth factor 20, is a neurotrophic factor preferentially expressed in the substantia nigra pars compacta. FGF20 can significantly improve the survival of cultured dopaminergic neurons via activating the MAPK pathway. FGF20 protects against the loss of dopaminergic neurons in the rat model of PD.^9^ Mounting evidence has indicated that variants in the *FGF20* gene were risk factors for PD in different ethnic populations.^10^, ^11^ Uladzislau Rudakou et al. found a novel rare variant (rs1034608171) located in the promoter region of the *FGF20* gene which drives the significant association with PD. However, this variant did not exist in our PD patients and healthy controls. The relatively smaller sample size or different ethnic context might have contributed to our failure in detecting this variate.

The GCH1 gene, which encodes GTP cyclohydrolase 1, belongs to the GTP cyclohydrolase family of enzymes known to be involved in the biosynthesis of tetrahydrobiopterin. Tetrahydrobiopterin is a cofactor for tyrosine hydroxylase that is a rate‐limiting enzyme for dopamine biosynthesis. Therefore, variants in the *GCH1* gene could cause dopa‐responsive dystonia.^12^ Moreover, many genetic association studies have also identified GCH1 variants that increased the risk for PD.^13^, ^14^ In our cohort, we only found one nonsynonymous variant (c.C552T, p.R186C) that exists in eight Parkinson's disease patients and four healthy controls. Moreover, analysis of conservation across different species and prediction of pathogenesis all suggested that the p.R186C variant may contribute to the pathogenesis of PD.

Taken together, our results indicated that the 10 variants in *SYT11*, *FGF20*, *GCH1* genes may not be causative variants for PD in eastern China. However, p.R186C variant likely increases the risk of PD. In consideration of the different results due to the diverse ethnic contexts, additional genetic association studies with larger samples and different ethnic cohorts are needed to identify the role of the variants in *SYT11*, *FGF20*, *GCH1* gene in PD.

## Supporting information

Table S1‐S2

## Data Availability

Data available on request from the authors.
