# Supplementary material for: Association analysis of SYT11, FGF20, GCH1 rare variants in Parkinson's disease
Source: CNS Neurosci Ther. 2021 Oct 21;28(1):175–7. doi: 10.1111/cns.13745 (PMC8673698; doi:10.1111/cns.13745)
Supplement: Supplementary file 1 — Table S1‐S2 [file CNS-28-175-s001.docx]

Supplementary table 1 Primers for MassARRAY assay.

| SNP ID or position | Forward primers | Reverse primers | UEP_SEQ |
| --- | --- | --- | --- |
| rs945006601 | ACGTTGGATGGCTGATAGCAAAAATACCCC | ACGTTGGATGTCTAGAGGTCTGCAGTAAGC | agggAGCCAAGAAACAAAGGTG |
| rs1034608171 | ACGTTGGATGATCCGGAACACAAAAGACCC | ACGTTGGATGGCCTTAACTTTTTGCGCTCG | tttgGGGATGGAGGTGGATAGA |
| 55312498 | ACGTTGGATGGCAGATGCAGACTTACGTTG | ACGTTGGATGGCTCCTTATCACATCCACAG | cCTGGAGTCGGGGTAG |
| 55312559 | ACGTTGGATGGCTCCTTATCACATCCACAG | ACGTTGGATGGCAGATGCAGACTTACGTTG | CTACAGCAATTTGTTTTGTAA |
| 55332087 | ACGTTGGATGAGATGGTGATTGTGAAGGAC | ACGTTGGATGTGCTGGGAAACAACAAAGAG | TGCTCACACATGGAAAA |
| rs104894434 | ACGTTGGATGGAACTCTTCCCGAGTCTTTG | ACGTTGGATGGTGTATGGTAATGCGAGGTG | ATGCGAGGTGTACAGAAAA |
| rs41298442 | ACGTTGGATGGTGTATGGTAATGCGAGGTG | ACGTTGGATGGAACTCTTCCCGAGTCTTTG | agTGTGCTGGTCACAGTT |
| rs756256944 | ACGTTGGATGTTGTCACAAAGAAGGCACTG | ACGTTGGATGGCCAAGGACTTGCTTGTTAG | gGTTAGGAAGATAACCAATATGGA |

Supplementary table 2 Primers for KASP assay.

| SNP ID or position | Primer 1 | Primer 2 | Primer common |
| --- | --- | --- | --- |
| 55312562 | TATCACATCCACAGTTCAGGAGC | CTTATCACATCCACAGTTCAGGAGT | GGCTTCCGTGATTGCTACAGCAATT |
| rs200891969 | AGACTTACGTTGCTTCAACCACTAC | CAGACTTACGTTGCTTCAACCACTAT | TTGCTGTAGCAATCACGGAAGCCTT |
